# Modulating the Behavior of Schwann Cells with NGF Exposure Combined with Different Energy Densities of Photobiomodulation Cultured on Polyhydroxybutyrate (PHB) Scaffolds

**DOI:** 10.3390/polym17212900

**Published:** 2025-10-30

**Authors:** Bryan Enoc Quidel-Necul, Paulina Martínez-Rodríguez, Karina Godoy Sanchéz, Glauce Crivelaro Nascimento, Bruna Balbino de Paula, Eduardo Borie, Fernando José Dias

**Affiliations:** 1 Master Program in Dental Sciences, Dental School, Universidad de La Frontera, Temuco 4780000, Chile; b.quidel01@ufromail.cl; 2 Doctorate in Applied Cellular and Molecular Biology, Universidad de La Frontera, Temuco 4780000, Chile; paulinaconstanza.martinez@ufrontera.cl; 3 Scientific and Technological Bioresource Nucleus (BIOREN), Universidad de La Frontera, Temuco 4780000, Chile; karina.godoy@ufrontera.cl; 4 Department of Oral and Basic Biology, School of Dentistry of Ribeirao Preto, University of Sao Paulo, Ribeirao Preto 14040-904, Brazil; glauce.nascimento@usp.br; 5 Department of Oral and Maxillofacial Surgery, College of Dentistry, University of Florida, Gainesville, FL 32610, USA; bbalbinodepaula@ufl.edu; 6 Department of Integral Adults Dentistry, Dental School, Universidad de La Frontera, Temuco 4780000, Chile; eduardo.borie@ufrontera.cl; 7 Research Centre in Dental Sciences (CICO-UFRO), Dental School, Universidad de La Frontera, Temuco 4780000, Chile; 8 Oral Biology Research Centre (CIBO-UFRO), Dental School, Universidad de La Frontera, Temuco 4780000, Chile

**Keywords:** Schwann cells, low-level laser, LLLT, nerve growth factor, NGF, polyhydroxybutyrate, PHB, scaffold, nerve regeneration, MTT assay crystal violet assay, SEM—Scanning Electron Microscopy

## Abstract

This study evaluated the effect of irradiation of different energy densities in low-level laser therapy (LLLT) and exogenous nerve growth factor (NGF) on Schwann cells (SCs). SCs (SCL 4.1/F7) exposed to LLLT (4 or 80 J/cm^2^) and NGF (25 ng/mL) were evaluated on days 1, 3, and 7. Cell viability (MTT), proliferation (crystal violet) and morphology (SEM—Scanning Electron Microscopy) on the polyhydroxybutyrate (PHB) scaffold were compared among five study groups: Control; L4. 4 J/cm^2^ LLLT; L80. 80 J/cm^2^ LLLT; L4N. 4 J/cm^2^ LLLT + NGF; and L80N. 80 J/cm^2^ LLLT + NGF. Viability and proliferation increased over time in groups treated exclusively with LLLT, with 4 J/cm^2^ reduced cell viability on the third day. The NGF exposition showed a reduction in cell viability and proliferation. The SCs remained attached to the PHB scaffold during the 7 days analyzed. The LLLT energy densities did not modify SC behavior, except for a reduction in cell viability after irradiation of 4 J/cm^2^ on the third day. Consistently, SC exposure to exogenous NGF significantly reduced proliferation and viability in all periods analyzed. Morphological changes were observed, and NGF exposure appears to have helped cells intertwine with PHB scaffold fibers.

## 1. Introduction

The nervous system is divided into a central nervous system (CNS) and a peripheral nervous system (PNS), where one of its basic cellular units is the neuron, which allows information to be sent and received from the entire human body. However, these need to be supported by different cell types that provide the neurons with physical, biological, and chemical protection [1]. Among these, we find the Schwann Cells (SCs), which are responsible for supporting the neurons and producing the myelin sheath, even guiding nerve regeneration within the PNS, and in some cases, given certain conditions, are also found in the CNS [2]. They are even used immersed in nerve guides to treat peripheral nerve injuries that have lost their continuity [3].

“LASER”, named because it is the acronym for “Light Amplification by Stimulated Emission of Radiation”, is a type of energy with the emission of photons with defined characteristics, among them monochromaticity (photons of light with a single color), coherence (the wavelength travels uniformly in time and space) and collimation (photons of light travel parallel to each other), among others. These characteristics allow a beam of photons with a defined wavelength to be emitted [4]. Lasers have been used in multiple areas of knowledge, including in the biomedical area, and can be classified in several ways: one of the most common is a high-intensity or surgical laser due to the ability to generate cuts and a low-level laser (LLLT), considered therapeutic [5]. The latter has the ability to stimulate different types of cells, producing a photobiomodulatory effect, but nevertheless, the ideal parameters for the use of LLLT to achieve a beneficial effect in different cells and especially in the use of SCs are not yet established, something which could improve the effects of nerve regeneration after peripheral nerve injury, achieving beneficial and reproducible results.

The use of LLLT has been the focus of attention in recent years; however, its use requires knowledge of several parameters, where we can find wavelength, power, intensity, irradiation time, and energy density, among others [6]. The energy density (dose) can be defined as the amount of energy accumulated in a region of space measured in “J/cm^2^” and has been one of the parameters of interest in recent years because its variation has been seen to cause the proliferation of SCs and modulation in the release of neurotrophic factors to a greater or lesser extent [7,8]. Within this topic, the densities most frequently used in the different studies have been 1 J/cm^2^, 4 J/cm^2^, 80 J/cm^2^, and 160 J/cm^2^ [6,9], where a large range of energy density utilization has been considered in the use of LLLT. On the other hand, histological and morphological evaluations have shown positive results in the use of LLLT in nerve regeneration [10,11,12].

Neurotrophic factors are secreted polypeptides that regulate survival, development, proliferation, migration, and differentiation at the cell membrane level to regulate various signaling processes acting on CNS and PNS cells. These factors not only play an important role in physiological activities but also in pathological processes [13]. Growth factors (including the neurotrophin family) are made up of Nerve Growth Factor (NGF), Brain-derived neurotrophic Factor (BDNF), Glial-cell Derived neurotrophic Factor (GDNF), neurotrophin-3 (NT-3), and neurotrophin (NT-4), each acting on specific receptors to exert their function [14,15].

Among the neurotrophic factors, NGF acts specifically on a subpopulation of small primary sensory neurons and sympathetic neurons. Peripheral nerve lesions induce cellular mechanisms similar to those activated during development, resulting in a rapid and robust increase in the synthesis of neurotrophic factors in neurons and SC, guiding and supporting regeneration [16,17,18].

Therefore, models for exploring the physiology and pathophysiology of SCs are of profound interest, especially for tissue engineering and clinical purposes, since they play a critical role in nerve regeneration upon injury and are affected in peripheral neuropathies [19,20].

Considering all of the above information, the objective of this research is to evaluate in an in vitro study with immortalized Schwann cells the effectiveness of photobiomodulation therapy (PBMT) with different energy densities in the use of LLLT associated with the neurotrophic factor NGF. The rationale for analyzing the combination of NGF and LLLT in Schwann cells lies in the fact that they are two different types of stimuli, LLLT being a physical stimulus and NGF a biological stimulus, that could generate a positive interaction, enhancing the effects of each other without competing for receptors in the cells. Furthermore, we found no reports in the scientific literature that have combined these stimuli directly on Schwann cells, thus favoring the scientific novelty of this study.

## 2. Materials and Methods

### 2.1. Schwann Cell Culture

The biosafety aspects of this study protocol were approved by the Scientific Ethical Committee of the Universidad de La Frontera approval no. 164_23 on 25 October 2023.

Commercial nerve Schwann cells acquired from Merck-Sigma (SCL 4.1/F7, catalog no. 93031204), from the “ECACC—European Collection of Authenticated Cell Cultures” and expanded from different passages, up to the fourth passage, were used. Cell assays used aliquots of 5000 and 100,000 cells [21,22]. The Schwann cells were thawed and then expanded into cell culture containers to cover at least 70% of the confluence area. Cells killed during expansion will be discarded by washing the container.

The live cells were detached from the container by trypsin, quantified, and then suspended in volumes with concentrations previously determined for use in culture plates with the base covered by scaffolds of polyhydroxybutyrate (PHB) microfilaments prepared using the electrospinning method, as previously described in Gutierrez et al. [22].

Schwann cells supplemented in a solution of Ham’s F-12 culture medium, 1% glutamine, 10% Fetal Bovine Serum (FBS), and 1% Penicillin–Streptomycin at 37 °C in a humidified atmosphere at 5% CO_2_ were monitored and maintained in culture vials on well-cut PHB sheets from a plate of 96 samples. The experiments were conducted in triplicate independently on all evaluation days on the 1st, 3rd, and 7th days [21,22].

The application of low-level laser therapy (LLLT) was performed on Schwann cells using the “Twin Laser” equipment (MMOptics, São Carlos, Brazil) with the 660 nm GaAlAs probe inside the laminar flow chamber using a black background that prevented the reflection of photons.

The experimental group was analyzed after the application of LLLT of 2 different energy densities, at 4 J/cm^2^ (40 mW, 4 s of irradiation) [6] and 80 J/cm^2^ (40 mW, 80 s of irradiation) [9], with or without associated neurotrophic factor. LLLT irradiation was performed after 1 day of cell seeding. These energy densities were chosen based on previous studies to analyze the behavior of SCs at a low dose of 4 J/cm^2^ [6] and a higher dose of 80 J/cm^2^ [9].

In this case, the neurotrophic factor used was the nerve growth factor (NGF) at a concentration of 25 ng/mL, which was added at the beginning of the experiment. The rationale for choosing the application of the exogenous NGF concentration was due to the fact that in SC cultures, the concentrations typically range from 10 to 50 ng/mL [23,24]. Therefore, 25 ng/mL lies within the commonly employed range, being sufficiently high to activate trophic receptors without reaching supraphysiological concentrations.

Thus, the groups of the present study were as follows:

- Control group: Schwann cells without exposure to LLLT and NGF.
- L4 group: Schwann cells irradiated with LLLT with 4 J/cm^2^.
- L80 group: Schwann cells irradiated with LLLT with 80 J/cm^2^.
- L4 + NGF group: Schwann cells exposed to LLLT with 4 J/cm^2^ and NGF (25 ng/mL).
- L80 + NGF group: Schwann cells exposed to LLLT with 80 J/cm^2^ and NGF (25 ng/mL).

The evaluations were carried out at 1, 3, and 7 days after the LLLT and NGF exposure.

### 2.2. Schwann Cell Viability—MTT Assay

Cell viability was evaluated between the different groups using the MTT assay. The MTT assay assesses the viability of the mitochondrial activity of the cells due to the formation of purple formazan crystals resulting from the reduction of a yellow tetrazolium salt (3-(4,5-dimethylthiazole-2-yl)-2,5-diphenyltetrazolium bromide or MTT) [25,26]. This procedure used the Tecan Infinite F50 plate reader with absorbance at 570 nm (TECAN, Männedorf, Switzerland) of the Center for Research in Oral Biology of the Faculty of Dentistry of the Universidad de La Frontera (CIBO-UFRO) [21,22].

### 2.3. Schwann Cell Proliferation—Crystal Violet Assay

The Crystal Violet assay is based on staining cells that adhere to cell culture dishes. After one wash step, the violet crystal dye is solubilized and measured through absorbance at 570 nm. The amount of staining with Crystal Violet in the assay is directly proportional to the cell biomass that is attached to the plaque. Violet crystal is a dye of triarylmethane that binds to ribose-like molecules such as DNA in nuclei [27].

This procedure was performed at CIBO-UFRO using Tecan Infinite F50 plate reader equipment (TECAN, Männedorf, Switzerland).

### 2.4. Cell Morphology, Morphometry, and Quantification—Scanning Electron Microscope Analysis

The morphology of Schwann cells was evaluated after 1, 3, and 7 days of incubation after the irradiation of LLLT and NGF exposure in the different groups in order to evaluate the morphological characteristics of the cells.

To do this, the cells were fixed in 2.5% glutaraldehyde (EMS, USA) in Sorensen’s phosphate buffer 0.1 M (Electron Microscopy Science, Hatfield, PA, USA) for 30 min, washed in ultrapure water, and taken to the Hitachi SU3500 scanning electron microscope (Hitachi, Tokyo, Japan) of the BIOREN-UFRO [21,22].

For the morphometric and quantitative analysis of CS prolongations, photomicrographs of SEM taken at a standard magnification of ×500 were used. With the aid of the ImageJ software (Version 1.54g—National Institutes of Health—NIH, Bethesda, MD, USA), the scale of the photo was standardized (Analyze > Set Scale) with the help of the tool to trace a line on the scale present in the photos obtained from the microscope. Subsequently, the extensions were measured with the help of the “segmented line” feature. The values obtained were stored in the form of a spreadsheet and later compared between the different groups in the same period and between the same group in different periods.

### 2.5. Data Analysis

The data collection was recorded in a Microsoft Office Excel spreadsheet. The statistical analysis of the data was carried out using the SigmaPlot 15.0 software (Grafiti, Palo Alto, CA, USA). Initially, the normality of the data was analyzed with the Shapiro–Wilk test. As the data did not present a normal distribution, in the case of comparisons of 3 groups, the Kruskal–Wallis test and the Tukey post hoc test were used. When comparing 2 groups, the Mann–Whitney test was applied. A value of *p* < 0.05 was chosen as the threshold for significance.

## 3. Results

### 3.1. Schwann Cell Viability—MTT Assay

**Figure 1:**
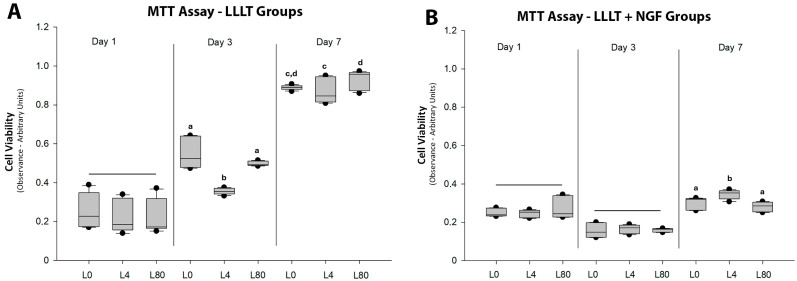
Cell viability (MTT assay). (**A**). LLLT groups without NGF. Bars indicate non-significant differences at day 1 (*p* > 0.05; L0 vs. L4 vs. L80, *p* = 0.122). Different letters within the same period denote statistically significant differences (*p* < 0.05): at day 3, a and b indicate significant differences (L0 vs. L4, *p* < 0.001; L0 vs. L80, *p* = 0.621; L4 vs. L80, *p* < 0.001); at day 7, c and d indicate significant differences (L0 vs. L4, *p* = 0.304; L0 vs. L80, *p* = 0.25; L4 vs. L80, *p* = 0.006). (**B**). LLLT + NGF groups. Bars indicate non-significant differences at day 1 and day 3 (*p* > 0.05; day 1: L0 + NGF vs. L4 + NGF vs. L80 + NGF, *p* = 0.825; day 3: L0 + NGF vs. L4 + NGF vs. L80 + NGF, *p* = 0.551). Different letters within the same period (day 7: a, b) denote significant differences (*p* < 0.05; L0 + NGF vs. L4 + NGF, *p* = 0.032; L0 + NGF vs. L80 + NGF, *p* = 0.128; L4 + NGF vs. L80 + NGF, *p* < 0.001).

The MTT assay in different analysis periods (1, 3, and 7 days—Figure 1A) of the cell groups that received only the application of LLLT revealed a gradual increase in viability over time. Significant changes were observed mainly after 3 days, with a reduction in the viability of Schwann cells that received 4 J/cm^2^ of irradiation.

The cell viability in different analysis periods (1, 3, and 7 days—Figure 1B) of the cell groups that received the application of NGF-associated LLLT (25 ng/mL) did not reveal an increase over time with different LRI irradiation energy densities. An increase in viability was only observed after 7 days in the L4 group compared to the other groups.

**Figure 2:**
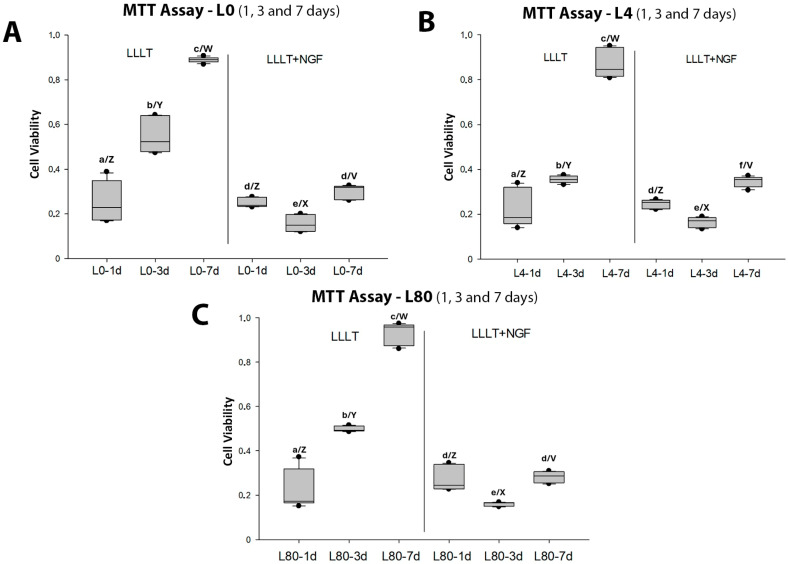
Comparison of Schwann cell viability (MTT assay) among LLLT and LLLT + NGF groups. (**A**). *Control group (L0)*, without and with NGF (25 ng/mL), evaluated at 1, 3, and 7 days. Different lowercase letters indicate significant differences (*p* < 0.05) between time points within the same protocol (L0: a–c; 1 d vs. 3 d *p* = 0.003, 1 d vs. 7 d *p* < 0.001, 3 d vs. 7 d *p* = 0.05; L0 + NGF: d–e; 1 d vs. 3 d *p* = 0.005, 1 d vs. 7 d *p* = 0.076, 3 d vs. 7 d *p* < 0.001). Different capital letters indicate significant differences (*p* < 0.05) between protocols at the same time point (L0 vs. L0 + NGF: 1 d *p* = 0.128; 3 d Y–X *p* < 0.001; 7 d W–V *p* < 0.001). (**B**). *L4 group* (LLLT at 4 J/cm^2^), with and without NGF (25 ng/mL), analyzed at 1, 3, and 7 days. Different lowercase letters indicate significant differences (*p* < 0.05) between time points within the same protocol (L4: a–c; 1 d vs. 3 d *p* = 0.005, 1 d vs. 7 d *p* < 0.001, 3 d vs. 7 d *p* = 0.04; L4 + NGF: d–f; 1 d vs. 3 d *p* = 0.015, 1 d vs. 7 d *p* = 0.015, 3 d vs. 7 d *p* < 0.001). Different capital letters indicate significant differences (*p* < 0.05) between protocols at the same time point (L4 vs. L4 + NGF: 1 d *p* = 0.128; 3 d Y–X *p* < 0.01; 7 d W–V *p* < 0.01). (**C**). *L80 group* (LLLT at 80 J/cm^2^), with and without NGF (25 ng/mL), evaluated at 1, 3, and 7 days. Different lowercase letters indicate significant differences (*p* < 0.05) between time points within the same protocol (L80: a–c; 1 d vs. 3 d *p* = 0.003, 1 d vs. 7 d *p* < 0.001, 3 d vs. 7 d *p* = 0.05; L80 + NGF: d–e; 1 d vs. 3 d *p* < 0.001, 1 d vs. 7 d *p* = 0.621, 3 d vs. 7 d *p* < 0.001). Different capital letters indicate significant differences (*p* < 0.05) between protocols at the same time point (L80 vs. L80 + NGF: 1 d *p* = 0.062; 3 d Y–X *p* < 0.001; 7 d W–V *p* < 0.001).

Comparison of the viability (MTT assay) of Schwann cells between the different groups (L0—Figure 2A, L4—Figure 2B, and L80—Figure 2C) with different protocols (without and with exposure to NGF 25 μg/mL) consistently showed that NGF exposure reduced cell viability regardless of LLLT energy density, especially after 3 and 7 days. In addition, it was observed that an increase in cell viability over time was negatively affected by the presence of NGF.

### 3.2. Schwann Cell Proliferation—Crystal Violet Cell Mass Assay

**Figure 3:**
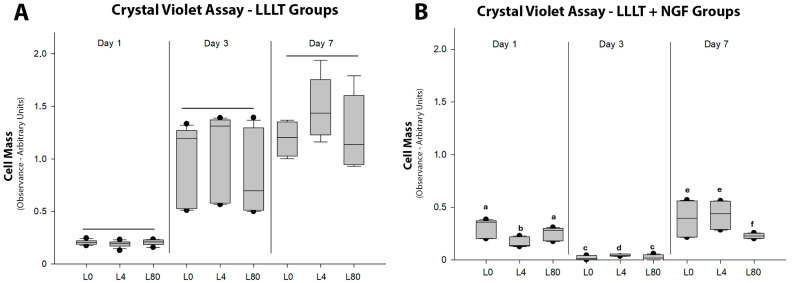
Cell proliferation (crystal violet assay). (**A**). *LLLT groups without NGF.* No statistically significant differences were observed among groups at any time point (*p* > 0.05; day 1: L0 vs. L4 vs. L80, *p* = 0.102; day 3: L0 vs. L4 vs. L80, *p* = 0.077; day 7: L0 vs. L4, *p* = 0.053; L0 vs. L80, *p* = 0.656; L4 vs. L80, *p* = 0.092). (**B**). *LLLT + NGF groups* (25 ng/mL). Different letters within the same period indicate significant differences (*p* < 0.05). Day 1: a, b (L0 + NGF vs. L4 + NGF, *p* < 0.001; L4 + NGF vs. L80 + NGF, *p* = 0.035). Day 3: c, d (L0 + NGF vs. L4 + NGF, *p* = 0.008; L4 + NGF vs. L80 + NGF, *p* = 0.048). Day 7: e, f (L0 + NGF vs. L80 + NGF, *p* = 0.018; L4 + NGF vs. L80 + NGF, *p* < 0.001).

The violet crystal assay in different analysis periods (1, 3, and 7 days) of the cell groups that received only the application of LLLT (Figure 3A) revealed a gradual increase in viability over time. No significant changes in Schwann cell proliferation were observed in any periods related to any specific energy density of the LLLT.

Cell proliferation was negatively affected after 3 and 7 days with exposure of Schwann cells to NGF (25 μg/mL, Figure 3B), and the gradual increase observed above was not observed. After 7 days, the energy density of 80 J/cm^2^ in the presence of NGF revealed the lowest values (*p* < 0.05) of Schwann cell proliferation.

**Figure 4:**
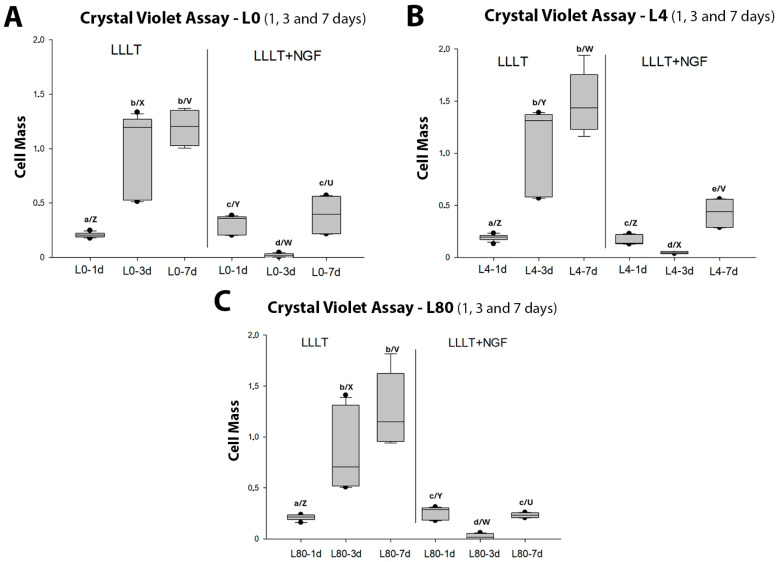
Comparison of cell proliferation (crystal violet assay) between LLLT and LLLT + NGF groups. (**A**). Control group (L0), without and with NGF (25 ng/mL). Different lowercase letters indicate significant differences between time points within the same protocol (*p* < 0.05; L0: a, b—1 d vs. 3 d *p* < 0.001, 1 d vs. 7 d *p* < 0.001; L0 + NGF: c, d—1 d vs. 3 d *p* = 0.002, 3 d vs. 7 d *p* < 0.001). Different capital letters indicate significant differences between protocols in the same period (*p* < 0.05; L0 vs. L0 + NGF—1 d *p* = 0.002; 3 d *p* < 0.001; 7 d *p* < 0.001). (**B**). L4 group (LLLT 4 J/cm^2^), with and without NGF (25 ng/mL). Different lowercase letters indicate significant differences within the same protocol (*p* < 0.05; L4: a, b—1 d vs. 3 d *p* < 0.001, 1 d vs. 7 d *p* < 0.001; L4 + NGF: c–e—1 d vs. 3 d *p* = 0.015, 1 d vs. 7 d *p* = 0.015, 3 d vs. 7 d *p* < 0.001). Different capital letters indicate significant differences between protocols in the same period (*p* < 0.05; L4 vs. L4 + NGF—3 d *p* < 0.01; 7 d *p* < 0.01). (**C**). L80 group (LLLT 80 J/cm^2^), with and without NGF (25 ng/mL). Different lowercase letters indicate significant differences within the same protocol (*p* < 0.05; L80: a, b—1 d vs. 3 d *p* < 0.001, 1 d vs. 7 d *p* < 0.001; L80 + NGF: c, d—1 d vs. 3 d *p* < 0.001, 3 d vs. 7 d *p* < 0.001). Different capital letters indicate significant differences between protocols in the same period (*p* < 0.05; L80 vs. L80 + NGF—1 d *p* = 0.028; 3 d *p* < 0.001; 7 d *p* < 0.001).

The crystal violet cell proliferation assay showed overall that regardless of the applied energy density of the LLLT (0—Figure 4A, 4 J/cm^2^—Figure 4B and 80 J/cm^2^—Figure 4C), Schwann cell proliferation was negatively affected by NGF exposure (25 ng/mL), especially after 3 and 7 days. Following the behavior of cell viability, there was no increase in cell proliferation over time.

### 3.3. Schwann Cell Morphology—Scanning Electron Microscopy

#### 3.3.1. Morphological Analysis—1 Day After LLLT Irradiation and NGF Exposure

**Figure 5:**
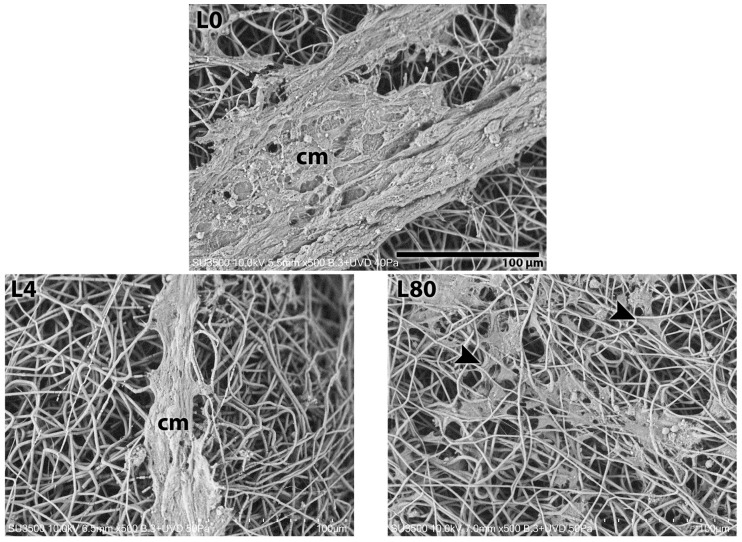

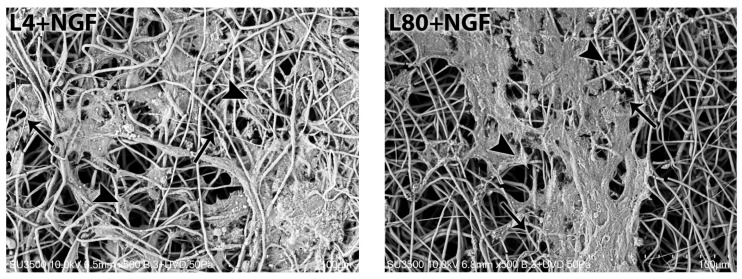
SEM analysis 1 day after irradiation with LLLT and NGF exposition. L0. Control group—without LLLT irradiation and no NGF exposition (Mag.: X500, Bar: 100 µm). L4. LLLT 4 J/cm^2^ group—LLLT irradiation with 4 J/cm^2^ (Mag.: X500). L80. LLLT 80 J/cm^2^ group—LLLT irradiation with 80 J/cm^2^ (Mag.: X500). L4 + NGF. LLLT 4 J/cm^2^ and NGF group—LLLT irradiation with 4 J/cm^2^ and NGF (25 ng/mL) exposure (Mag.: X500). L80 + NGF. LLLT 80 J/cm^2^ and NGF group—LLLT irradiation with 80 J/cm^2^ and NGF (25 ng/mL) exposure (Mag.: X500). Figure tags: Schwann cell mass (cm), SC (arrowhead), and thin filaments (thin arrow).

After 1 day of LLLT irradiation and NGF exposure, the presence of Schwann cells attached to the PHB scaffold was observed in all the groups analyzed, with morphological differences. The control groups (without LLLT and NGF—Figure 5(L0)) and L4 (Figure 5(L4)) revealed more concentrated elongated Schwann cell masses. The groups L80 (Figure 5(L80)), L4 + NGF (Figure 5(L4 + NGF)), and L80 + NGF (Figure 5(L80 + NGF)) revealed adherent cells with a stellate and elongated shape and intercalated with the PHB fibers. The presence of very thin filaments is also observed in the sample of the L4 + NGF and L80+NGF groups, suggesting cellular connections.

#### 3.3.2. Morphological Analysis—3 Days After LLLT Irradiation and NGF Exposure

**Figure 6:**
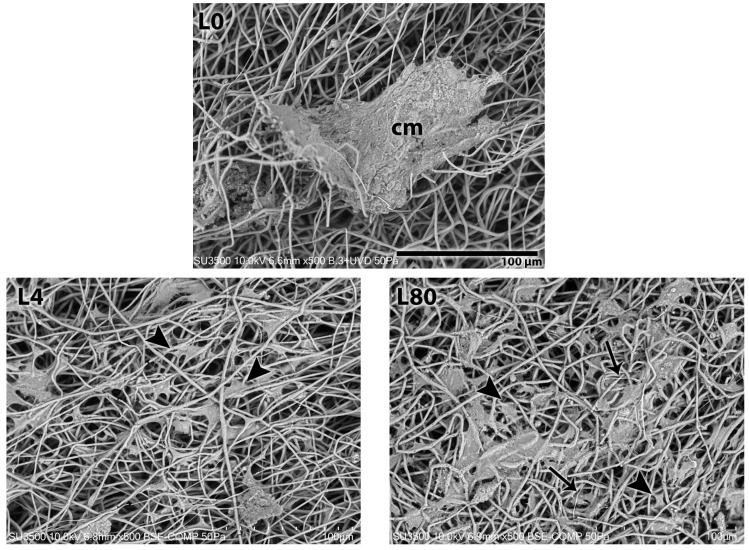

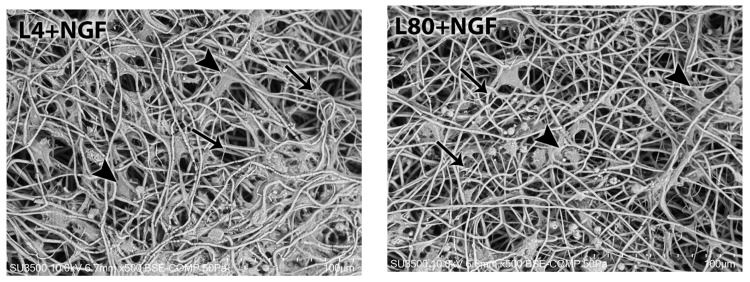
SEM analysis 3 days after irradiation with LLLT and NGF exposition. L0. Control group—without LLLT irradiation and no NGF exposition (Mag.: X500, Bar: 100 µm). L4. LLLT 4 J/cm^2^ group—LLLT irradiation with 4 J/cm^2^ (Mag.: X500). L80. LLLT 80 J/cm^2^ group—LLLT irradiation with 80 J/cm^2^ (Mag.: X500). L4 + NGF. LLLT 4 J/cm^2^ and NGF group—LLLT irradiation with 4 J/cm^2^ and NGF (25 ng/mL) exposure (Mag.: X500). L80 + NGF. LLLT 80 J/cm^2^ and NGF group—LLLT irradiation with 80 J/cm^2^ and NGF (25 ng/mL) exposure (Mag.: X500). Figure tags: Schwann cell mass (cm), SC (arrowhead), and thin filaments (thin arrow).

After 3 days of LLLT and NGF applications, the presence of Schwann cells adhering to the PHB scaffold was observed, in the PHB scaffold of all the groups analyzed. Again, the control group (Figure 6(L0)) revealed a more compact cell mass. The other groups treated with LLLT with or without association with NGF (L4—Figure 6(L4), L80—Figure 6(L80), L4 + NGF—Figure 6(L4 + NGF) and L80 + NGF—Figure 6(L80 + NGF)) revealed a large number of cells intercalated with PHB filaments with a more star-like shape compared to the control group and the previous analysis period. Once more, very thin filaments are observed, especially in the L80, L4 + NGF, and L80 + NGF groups, suggesting cellular connections.

#### 3.3.3. Morphological Analysis—7 Days After LLLT Irradiation and NGF Exposure

**Figure 7:**
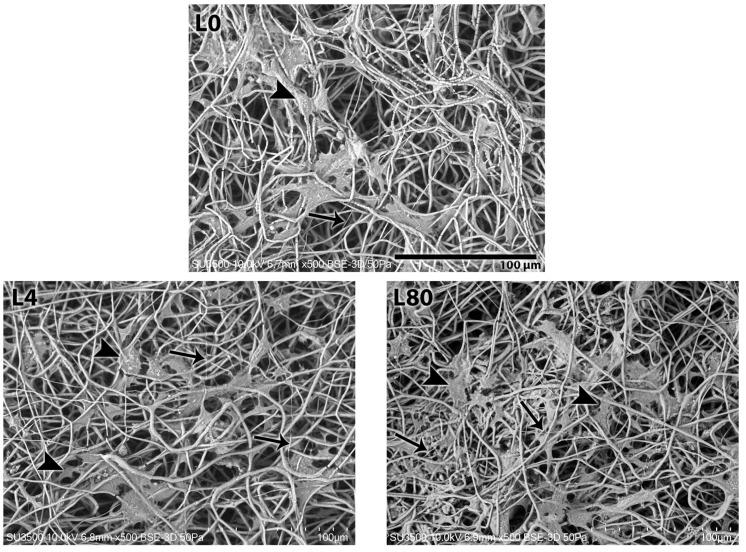

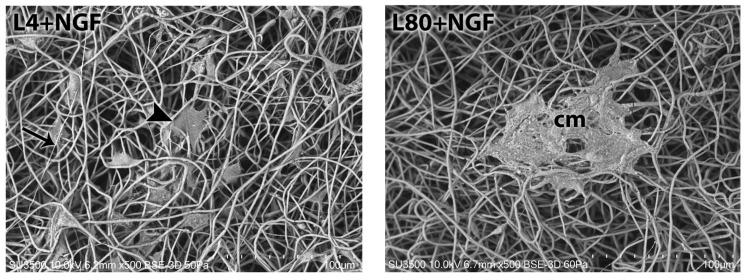
SEM analysis 7 days after LLLT irradiation and NGF exposition. L0. Control group—without LLLT irradiation and no NGF exposition (Mag.: X500, Bar: 100 µm). L4. LLLT 4 J/cm^2^ group—LLLT irradiation with 4 J/cm^2^ (Mag.: X500). L80. LLLT 80 J/cm^2^ group—LLLT irradiation with 80 J/cm^2^ (Mag.: X500). L4 + NGF. LLLT 4 J/cm^2^ and NGF group—LLLT irradiation with 4 J/cm^2^ and NGF (25 ng/mL) exposure (Mag.: X500). L80 + NGF. LLLT 80 J/cm^2^ and NGF group—LLLT irradiation with 80 J/cm^2^ and NGF (25 ng/mL) exposure (Mag.: X500). Figure tags: Schwann cell mass (cm), SC (arrowhead), and thin filaments (thin arrow).

After 7 days of LLLT irradiation and NGF exposure, the presence of Schwann cells was still observed in all the samples analyzed. All groups, except L80 + NGF (Figure 7(L80 + NGF)), revealed cells attached to and interspersed with PHB fibers in a stellate shape.

In the control group (Figure 7(L0)), L4 (Figure 7(L4)), L80 (Figure 7(L80)), and L4 + NGF (Figure 7(L4 + NGF)), thin filaments that suggest cellular connections can be observed. During this period, the onset of PHB fiber degradation is evident, observed in the control group. The L80 + NGF group (Figure 7(L80 + NGF)) revealed a more compact cell mass.

The data and analysis of the measurements performed on the Schwann cell extensions are presented in Table 1.

On day 1, the presence of SC prolongations was noted mainly in the L80, L4 + NGF and L80 + NGF groups. In group C, these prolongations were not observed. The count reveals an increase in the number of prolongations, mainly from 1 to 3 days, in this period all groups showed prolongations, and the groups treated with LLLT and NGF showed the highest values. After 7 days, group C, unlike in previous periods, revealed a high number of SC prolongations.

The comparison of the mean lengths of the prolongations of different groups in the same period revealed a significant difference (*p* < 0.001) only after 3 days, with the L4 group presenting the longest prolongations. The other groups in this period and in the other periods did not show significant differences.

The comparison of the prolongation sizes of the same group in different periods revealed that the L80 group after 3 days was significantly smaller than the values observed after 1 day (*p* = 0.039) and after 7 days (*p* = 0.013). In addition, the L4 + NGF group had significantly greater prolongations (*p* < 0.001) after 7 days compared to the prolongations of this group after 3 days.

The sum of all the extensions analyzed reveals in general that the values increase over time in all groups.

In general, there is no clear trend in the behavior of the minimum and maximum sizes of the extensions analyzed. The sizes measured were from 4.7 to 205.3 µm.

## 4. Discussion

PBMT has been used for more than 30 years to treat neurological diseases. Low-level lasers are commonly used for clinical applications. The growing interest in the application of this type of laser in neuron-related diseases is due to the absorption of photons by intracellular structures; the effect on the oxidative state of cells; and the effect on the expression of proteins involved in oxidative stress, inflammation, pain, and neuronal growth [28].

PBMT has been investigated because of its close relationship with peripheral nerve damage. The wavelength of infrared irradiation is easily absorbed by tissues, and the loss of intensity is minimal, affecting metabolic modifications, DNA activity, adenosine triphosphate (ATP) formation, and the mitochondrial chain. The effect of photobiomodulation is due to the absorption of photons by cytochrome C oxidase in the mitochondrial respiratory chain, thereby increasing cytochrome C oxidase activity and thus ATP formation [29]. ATP from injured regions or altered blood perfusion can reactivate injured cells and metabolic disorders. However, PBMT presents difficulties in selecting the most appropriate parameters for its application due to the lack of standardization since wavelength, power density, irradiation time, and polarization of light have an impact on biological effects [30].

The present study analyzed the in vitro behavior of Schwann cells, glial cells of the PNS crucial in peripheral nerve regeneration [2], with applications of two different energy densities (doses) of PBMT with LLLT associated with exposure to NGF, the main neurotrophic factor associated with nerve regeneration [16,17].

Our results revealed that in general, in the groups treated only with PBMT, the viability and proliferation of SCs increased over time in the periods analyzed (1, 3, and 7 days).

The two energy densities of LLLT (low of 4 J/cm^2^ and high of 80 J/cm^2^) not associated with NGF exposure (25 ng/mL) did not alter cell behavior. The only exception was the dose of 4 J/cm^2^ after 3 days of irradiation, which negatively affected viability. SC exposure to exogenous NGF (25 ng/mL) revealed a consistent behavior of reduction in cell viability and proliferation values. In these groups (L4 + NGF and L80 + NGF) the values of these assays remained low in all analysis periods (1, 3, and 7 days), thus differing from the behavior of cells treated only with LLLT.

SCs are crucial for maintaining the homeostasis of peripheral nerves via the secretion of NGF. The mTOR signaling pathway is known as a regulator of various cell functions; and the DNA methyltransferase 1 (DNMT1) is an epigenetic regulator and downstream target of the mTOR pathway. DNMT1 is the downstream target of the mTOR pathway and mediates the mTOR pathway inhibition-induced reduction in NGF expression in Schwann cells [31]. The exogenous application of 25 ng/mL of NGF could have activated DNMT1 and thus may have reduced the effects of endogenous NGF via mTOR pathway. Another hypothesis is that there may have been negative feedback in which SCs may have reduced its viability and proliferation due to the presence of NGF in the culture medium.

Previous studies have demonstrated that when mitochondrial function is compromised in SCs, failures occur in the respiratory chain, leading to decreased ATP production and consequent metabolic dysfunctions [32]. Dysfunctional mitochondria also increase the generation of reactive oxygen species (ROS), which exert oxidative damage on lipids, proteins, and cellular DNA, resulting in a ROS burst that may trigger the activation of cell death pathways [33]. The combination of low ATP levels (insufficient energy) and high ROS production (oxidative damage) places SCs at risk of losing their functionality or viability, for example, through impaired maintenance of axons or myelin. That could be another explanation for the results observed in the present study.

After peripheral nerve injury (PNI), growth factors, such as NGF, play an important role in promoting cell growth and survival, axon and myelin sheath regeneration, cell differentiation, and angiogenesis. In this context NGF is upregulated in SCs, that supports the survival and sprouting of neurons expressing Trk A. In addition, increased levels/expression of NGF and the p75 receptor can stimulate SC proliferation and migration and regulate SC apoptosis [34,35,36,37].

The PI3/KAkt signaling pathway is a main molecular mechanism for regulating peripheral nerve regeneration, activated by NGF that regulates processes such as cell proliferation, differentiation, apoptosis, and migration. The MAPK/ERK pathway is another signaling pathway related to peripheral nerve regeneration. It contributes to the survival of injured neurons, axon growth, and the myelination of regenerating axons [34,38,39]. NGF in the treatment of PNI stimulates the proliferation, migration, and differentiation of SCs; regulates the differentiation of SC phenotypes; and promotes the myelination of nerve fibers [34,40,41,42].

Administration of exogenous NGF could activate autophagy in dedifferentiated SCs, accelerate myelin debris clearance and phagocytosis, as well as promote axon and myelin regeneration at early stage of PNI. These NGF effects were effectively blocked by autophagy inhibitors. NGF effect on promoting early nerve regeneration is closely associated with its accelerating autophagic clearance of myelin debris in SCs, which probably regulated by the p75NTR/AMPK/mTOR axis [40].

Although few studies explicitly demonstrate that exposure of SCs to exogenous nerve growth factor (NGF) directly reduces cell viability, several findings suggest that adverse effects may occur under certain conditions. Soilu-Hänninen et al. [43] showed that SCs transfected with the anti-apoptotic gene Bcl-2 remained susceptible to apoptosis through activation of the low-affinity receptor p75^NTR, indicating that NGF can trigger death pathways in addition to survival signaling. These effects appear to depend on the dose and duration of exposure. The neurotrophic factor responses in SCs and neurons are highly influenced by concentration, exposure time, and interactions with other stimuli [44]. Therefore, although NGF generally promotes cell survival and differentiation, its prolonged or high-dose application—especially in combination with other stimuli such as low-level laser therapy (LLLT)—may induce apoptotic signaling and reduce SC viability through p75^NTR-mediated mechanisms. Thus, careful control of NGF concentration and exposure time is essential to prevent potential cytotoxic or pro-apoptotic effects in experimental and therapeutic contexts.

The association of LLLT with exogenous NGF exposure showed that the energy density of 80 J/cm^2^ reduced proliferation after 7 days compared to the other groups; and that the dose of 4 J/cm^2^ increased viability at 7 days, with values similar to the first day.

The morphological analysis of the SEM showed the presence of SCs adhered to the fibers of the PHB scaffold in all groups in the 3 periods analyzed (1, 3, and 7 days). Differences in morphology related to the exposure of the different energy densities of LLLT and exogenous NGF (25 ng/mL) were noted.

After one day, all groups exhibited SC attachment, confirming PHB biocompatibility, while those exposed to LLLT—particularly with NGF—showed elongated and stellate morphologies with thin cytoplasmic projections intercalating between fibers, suggesting early cytoskeletal remodeling and intercellular connection formation. By the third day, these features were more evident, especially in groups treated with LLLT and NGF, where cells appeared more dispersed and integrated with the scaffold. After seven days, all groups maintained viable cells on the scaffold, confirming PHB’s stability during early degradation. Stellate morphologies persisted, but the L80 + NGF group displayed a more compact cell mass, possibly indicating overstimulation or feedback regulation at higher doses of 80 J/cm^2^.

The presence of prolongations can help to understand the establishment of networks between SCs on the PHB scaffold with different stimuli in the different periods analyzed. The morphometric and quantitative analysis performed in the present study of these extensions suggest behaviors with the protocols and periods evaluated.

In general, the number of prolongations seems to have increased over time in all groups analyzed, especially between 1 and 3 days. It is noteworthy that the groups treated with LLLT with or without the combination with NGF revealed the presence of this prolongation from day to day 7, being more evident in the L80, L4 + NGF and L80 + NGF groups from day 1.

The mean size of the SC prolongations, although they revealed significant differences such as between the L4 group after 3 days and the other groups of that period and the L80 and L4 + NGF groups in different periods, do not seem to reveal a pattern or trend related to the stimuli and periods analyzed. However, they corroborate the behavior of the L4 viability trial after 3 days compared to the other groups in this period; and the L4 + NGF group between 3 and 7 days.

The sum of the prolongations agrees with the increase in the quantified number, in which in general the values increased in all groups in different periods analyzed. The control group stands out, which after 1 day did not reveal the presence of these prolongations and at the end of 7 days already presented values of the sum of the prolongations that were more similar to the other groups.

Finally, the minimum and maximum size values of the SC prolongations also did not reveal any clear trend or pattern. The high variability of the measured sizes is noteworthy, which can be observed in the high values of the standard deviations of the groups, and the size range from 4.7 to 205.3 μm.

After a peripheral nerve injury, SCs initiate a repair program characterized by extensive cell elongation and intense branching, forming long parallel-aligned extensions that act as support for axonal regeneration [45]. The development of these prolongations depends on reciprocal interactions between axons and SCs mediated by extrinsic signals from both axons and ECM. These stimuli influence the fate of SCs and promote the cytoplasmic reorganization necessary to form the prolongations [46]. Molecules such as Neuregulin 1 (NRG1), originating from the axon, interact with receptors in the SCs, triggering signaling pathways that coordinate cell behavior, and may contribute to the formation of extensions [47].

Although there is no consensus in the literature that NGF directly stimulates the formation of prolongations in SC, there are indications of mechanisms by which it can modulate morphology, migration, adhesion and cellular plasticity that would favor the extension of cellular processes.

One mechanism that could help explain this relationship would be the expression of NGF receptors by SC. The SCs express the p75^NTR receptor at various stages, including after injury [48]. Activation of the p75^NTR receptor by NGF can trigger intracellular signaling cascades that modulate cytoskeleton, cell morphology, migration, and adhesion [49,50]. The activation of the p75^NTR receptor by NGF in CS could also be associated with the activation of the Src/Vav2 cascade, a regulator of Rho-GTPases that are related to the reorganization of the cytoskeleton through the alteration of the dynamics of actin and microtubules, essential for the formation of cell extensions [51].

The limitations and gaps in the literature on the relationship between the formation of SC prolongations associated with NGF are due to the few studies that document and analyze the formation of these prolongations. In addition to the dependence on the context of these studies, it may depend on phenotypic cell expression, the presence of other signaling molecules, different concentrations of NGF, the cell receptors involved, and the complexity of cross signaling of different pathways, which makes it difficult to understand this interaction.

A cell mass was observed in the control group on days 1 and 3, in the L4 group on day 1, and in the L80 + NGF group after 7 days. In the other groups, the cells were better distributed and interspersed with PHB fibers in the L80 (1, 3, and 7 days), L4 + NGF (1, 3, and 7 days), and L80 + NGF (1 and 3 days) groups in the different analysis periods.

Finally, the presence of fine filaments, suggesting cellular connections, was observed from day 1 in the NGF groups (L4 + NGF and L80 + NGF), after 3 days in the groups (L80, L4 + NGF, and L80 + NGF) and finally after 7 days in the control groups, L4, L80, and L4 + NGF.

Currently, although there are many studies carried out in animal models that report the effects of the application of PBMT on nerve regeneration considering Schwann cells with positive results, such as faster and more efficient nerve recovery (904 nm, 4 J/cm^2^) [52]; acceleration of functional recovery and improvement of the quality of nerve regeneration after autograft repair (810 nm, 25 J/cm^2^) [53]; improvement of motor functions and electrophysiological reactions, reduction in muscle atrophy, and morphometric recovery (660 nm) [54]; increased number of SCs, large myelin axons, and neurons (680 nm, 10 J/cm^2^) [55]; significant improvement in nerve morphometry (904 nm, 6 J/cm^2^) [56] and improvement in motor recovery (780 nm, 15 J/cm^2^) [57], great variability in PBMT application parameters is observed, especially in relation to energy/dose density (J/cm^2^) and wavelength (nm).

For this reason, there are in vivo studies that are already concerned with reporting different results of nerve regeneration with different LLLT parameters, in which energy densities of 10 and 60 J/cm^2^ (660 nm) accelerated neuromuscular recovery compared to the density of 120 J/cm^2^ (780 nm) [11]. Improvement in recovery from nerve injuries with different energy densities of 4, 10, and 50 J/cm^2^ (780 nm) and the best morphometric results were observed at 15 days with a density of 10 J/cm^2^ [8]. The wavelengths of 633 and 780 nm inhibited the death of geniculate ganglion neurons, but the wavelength of 804 nm did not [58].

As in animal studies, in vitro studies with SCs reveal a high degree of heterogeneity in the studies in relation to the parameters used with LLLT, wavelengths of 632.8, 810, 660, and 780 nm, powers of 5.98, 40, and 50 mW and energy densities of 1, 4, 80 and 160 J/cm^2^. There is also a lack of agreement in relation to the parameters analyzed, the most common being cell viability and proliferation analyses [6,9,59], data that were also analyzed in the present study.

In this study, the energy density of 4 J/cm^2^, without the presence of NGF, showed the lowest viability values on 3rd day. And only after 7 days, the viability of the group irradiated with 4 J/cm^2^ was similar to that of the other groups. However, in the presence of NGF, the group irradiated with 4 J/cm^2^ showed the highest viability values after 7 days. These results were similar to those found by Yazdani et al. [6], who observed a decrease in SC proliferation measured through the MTT test with the application of an energy density of 4 J/cm^2^ on the fourth day of evaluation and then a growth trend on day 7 of evaluation, and this could be due to the elimination of the “stress” provided by the “dose of energy” applied by the LLLT to the SCs.

However, none of these studies looked at the association of PBM/LLLT with exogenous NGF exposure as was performed in our study. Previous studies reveal that NGF-regulated axonal signals have opposite effects on the central and peripheral nervous systems by promoting myelination in the SCs of PNS and reducing CNS oligodendrocytes [60]. Another possible action of NGF on SCs would be to activate autophagy for the removal of myelin debris resulting from nerve injury [40]. These changes in the phenotypes and behaviors of SCs in the face of NGF exposure could help explain the observed reduction in both cell viability and proliferation observed in the results of the present study. However, factors related to the myelination or autophagic capacity of SCs were not analyzed.

In this context, future S100β and P0 labeling could help improve the understanding of CS phenotypes. The S100β marker is recognized as an indicator of glial lineage in SCs and may reflect their state of maturation or activation [19]. Mata et al. [61] have shown that the degree of immunoreactivity for S100 correlates with the thickness of the myelin sheath formed by SCs; that is, more active or more mature SCs undergoing myelination exhibit higher S100β expression. The P0 marker (also known as MPZ—Myelin Protein Zero) is a structural component of the myelin sheath in the peripheral nervous system and has been used to infer Schwann cell (SC) differentiation toward a myelinating phenotype. A classical study demonstrated that SCs from transected rat nerves synthesized P0 basally, whereas neonatal SCs lost this expression in culture, suggesting that newly generated SCs revert to a precursor state when P0 expression declines [62]. More recently, in SC “repair” models, the simultaneous expression of S100β and P0 has been used to characterize the state of cells after injury or in culture [63]. Therefore, elevated levels of S100β may indicate SCs in an activated or differentiating state, whereas lower levels or absence of this marker could suggest a more quiescent condition or a phenotype less committed to myelination. The presence of P0 indicates that SCs are progressing toward functional differentiation for axonal support and myelination, while the absence or low expression of P0 may reflect an immature or quiescent state.

Polyhydroxybutyrate (PHB) is a biodegradable biopolyester capable of significantly increasing cell adhesion, proliferation and, regeneration, and it is for this reason that it is used as nerve guidance or scaffolds [64] thus allowing a growth pathway for SCs to be observed in scanning electron microscopy. The morphological changes observed in our studies related to the different treatments applied should be highlighted, and it was possible to observe viable cells adhered to the fibers of the PHB scaffold up to 7 days after cell treatments.

Andreo et al. [9] observed that the SCs showed a morphology similar to the neuronal cell with normal cytoskeleton organization, results similar to those found in this study, where stellate cells were also observed with the formation of fine interconnected filaments in some cases. In animal research, the study of cell morphology with the application of LLLT has not been exclusive to SC, neuronal cells would show a better distribution and organization of nerve fibers when applying LLLT after nerve injury [8], as well as an increase in the thickness of the myelin sheath [10] where also Schwann cells would show an apparently reactive nucleus with characteristics of synthesis activity and signs of neuronal regeneration [55].

Due to the few studies and the heterogeneity of PBMT parameters, more studies are still needed on the behavior and modulability of SCs in the face of different types of stimuli, such as PBMT with different parameters and the application of neurotrophic factors, in order to better understand the therapeutic possibilities and biological mechanisms related to peripheral nerve regeneration.

Although morphological analysis with SEM allows a three-dimensional analysis of the interaction of cells with PHB’s scaffold, it should be noted that this method of analysis has limitations in the analysis of the general distribution and quantification of cells on the material, mainly by seeding the cells on the material that, because it is standardized and more systematized, it will always be irregular. The limitation of the morphometric and quantitative analysis performed on the photomicrographs obtained by SEM-VP, especially with regard to the costs of preparing an adequate number of samples and the difficulty of their standardization. In addition to the difficulty of differentiating cell structures compared to PHB scaffold structures, in which future cytoskeletal staining could help. Even so, the authors did their best, standardizing the analyses on the material obtained using a methodology and increases determined for each group analyzed in each period.

In the future, consideration should be given to expanding the analysis primarily to factors related to myelin production and the expression of the quiescent, autophagic or myelinating phenotypes of SCs. In addition, other concentrations of exogenous NGF should be considered for a better analysis of its interaction with SC behavior.

## 5. Conclusions

Partial modulation of SCs was observed with different viability, proliferation, and morphology behaviors, compared to cellular exposure to different energy densities of PMBT/LLLT (4 and 80 J/cm^2^) in different periods. However, exposure to NGF (25 ng/mL) did not improve outcomes; on the contrary, this exposure systematically reduced cell viability and proliferation.

PBMT/LLLT energy density of 4 J/cm^2^ with no NGF association reduced cell viability after 3 days. The association of exogenous NGF (25 ng/mL) showed a consistent response of reduced cell viability and proliferation, regardless of the association with different LLLT energy densities.

The cells remained attached to the fibers of the PHB scaffold during the 7 days analyzed. Notably, after 1 day, cells interspersed with PHB fibers were already observed in the LLLT- and NGF-treated groups.

Considering the importance of understanding the possibilities of modulating the behavior of SCs, mainly related to the regeneration of peripheral nerves; the maturation, growth, and development of the PNS; and diseases related to demyelination and the small number of studies of the interaction of these cells with PBMT/LLLT, and associated with other factors, more studies are needed to understand more and more the behavior of these cells and thus be able to seek treatment alternatives for the problems associated with the PNS.

## Figures and Tables

**Table 1:** Morphometry and quantification of Schwann cell extensions.

Group	Counting (Number)	Mean ± SD (μm)	Sum (μm)	Min. (μm)	Max. (μm)
C-1d	0	0	0	0	0
L4-1d	7	61.5 ± 62.0	492.0	18.8	188.3
L80-1d	32	44.2 ± 26.3	1415.6	8.8	141.4
L4 + NGF-1d	40	33.4 ± 21.0	1335.7	7.3	99.5
L80 + NGF-1d	42	40.2 ± 29.3	1686.2	8.9	142.1
C-3d	10	19.4 ± 11.4	193.7	4.7	44.2
L4-3d	42	53.9 ± 37.1 *	2262.4	8.3	179.9
L80-3d	35	29.5 ± 17.9 ^a^	1033.3	5.7	64.9
L4 + NGF-3d	94	27.2 ± 16.7	2556.4	7.8	105.7
L80 + NGF-3d	69	30.5 ± 18.8	2104.7	10.6	91.8
C-7d	61	34.4 ± 21.6	2098.0	8.0	133.2
L4-7d	61	49.14 ± 32.5	2997.3	5.2	177.9
L80-7d	79	51.1 ± 40.4	4034.4	6.8	205.3
L4 + NGF-7d	72	43.5 ± 26.8 ^b^	3129.7	9.9	145.7
L80 + NGF-7d	48	43.4 ± 36.9	2082.4	7.7	164.1

* = L4-3d higher than the other 3-day groups (*p* < 0.001). ^a^ = L80-3d lower than L80-1d (*p* = 0.039) and L 80-7d (*p* = 0.013). ^b^ = L4 + NGF-7d higher than L4 + NGF-3d (*p* < 0.001).

## Data Availability

The original contributions presented in this study are included in the article. Further inquiries can be directed to the corresponding author.

## Abbreviations

The following abbreviations are used in this manuscript:

NGF	Nerve growth factor
SC	Schwann cell
LLLT	Low-level laser therapy
PBMT	Photobiomodulation therapy
PHB	Polyhydroxybutyrate
L0	Control group—LLLT 0 J/cm^2^
L4	LLLT 4 J/cm^2^ study group
L80	LLLT 80 J/cm^2^ study group
L4 + NGF	LLLT 4 J/cm^2^ & NGF (25 ng/mL) study group
L80 + NGF	LLLT 80 J/cm^2^ & NGF (25 ng/mL) study group
